# Statin diabetogenicity: guidance for clinicians

**DOI:** 10.1186/1475-2840-12-S1-S3

**Published:** 2013-05-30

**Authors:** Kausik Ray

**Affiliations:** 1 St George’s Hospital, University of London, Cranmer Terrace, London SW17 0RE, UK

## Abstract

Type 2 diabetes (T2D) is a strong, independent risk factor for cardiovascular (CV) and cerebrovascular outcomes. Meta-analysis of five randomised clinical trials (*n* = 33,040) showed that, although intensive versus standard glycaemic control significantly reduced CV events in people with T2D, the reduction was less than that achieved with lipid-lowering or antihypertensive treatment. Furthermore, fasting plasma glucose (FPG) concentrations were a modest predictor for CV risk in people without T2D. Thus, although effective glycaemic control is important for the prevention/management of T2D, other risk factors must be addressed to effectively reduce CV risk. Reducing low-density lipoprotein-cholesterol levels using statins significantly reduces CV risk in people with and without T2D. Although statins are generally safe and well tolerated, conflicting data exist regarding the diabetogenic effects of some statins. Based on recent clinical trial data, the US Food and Drug Administration have changed the labelling of all statins to include ‘an effect of statins on incident diabetes and increases in haemoglobin A1c and/or FPG’. However, the literature suggests that the beneficial effects of most statins on CV risk continue to outweigh their diabetogenic risks and that statins should remain as first-line therapy for the majority of people with dyslipidaemia and metabolic syndrome or T2D. Mechanisms explaining the potentially higher incidence of T2D with statin therapy have not been confirmed. However, independent predictors for statin-associated T2D appear to include elevated levels of baseline FPG, BMI, blood pressure and fasting triglycerides. Moreover, although some statins (for example, atorvastatin) are associated with increased haemoglobin A1c levels in patients receiving intensive but not moderate therapy, other statins (for example, pitavastatin) have demonstrated neutral or favourable effects on glucose control in patients with and without T2D or metabolic syndrome. The potential diabetogenic effects of statins may therefore differ between drugs. In conclusion, conflicting data exist regarding the diabetogenic effects of statins. Further studies are required to understand whether all statins have the same effect and whether some patient groups are at higher risk than others. Meanwhile, results suggest that the net CV benefit favours the use of statin therapy in patients with dyslipidaemia, irrespective of T2D risk.

## Introduction

**Figure 1:**
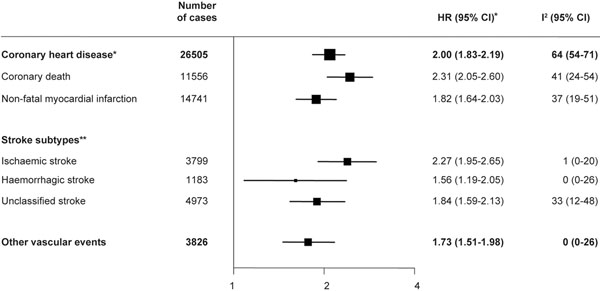
**Type 2 diabetes confers excess risk for a wide range of vascular diseases.** *Adjusted for age, smoking, BMI, systolic blood pressure and stratified by sex and trial arm (where appropriate). **lncludes fatal and nonfatal events.

Type 2 diabetes (T2D) is a well-established risk factor for cardiovascular (CV) and cerebrovascular disease [1-3]. However, the extent to which its impact on vascular risk varies according to levels of conventional risk factors was, until recently, unknown. A meta-analysis of 102 prospective studies (*n* = 698,782) from the Emerging Risk Factors Collaboration demonstrated that T2D confers approximately a twofold excess risk for a wide range of vascular diseases, including coronary heart disease (CHD; hazard ratio (HR) = 2.00, 95% confidence interval (CI) = 1.83 to 2.19), ischaemic stroke (HR = 2.27, 95% CI = 1.95 to 2.65), haemorrhagic stroke (HR = 1.56, 95% CI = 1.19 to 2.05), and other vascular deaths (HR = 1.73, 95% CI = 1.51 to 1.98) (Figure 1) [2]. T2D was a strong predictor for CV risk in all patient subgroups, and was more strongly associated with fatal versus nonfatal disease. HRs were largely unaffected by inflammatory/ renal markers and the lipid profile (non-high-density lipoprotein, high-density lipoprotein and triglyceride levels). This study demonstrates that the association between T2D and vascular disease cannot fully be explained by conventional or emerging CV risk factors and suggests that the causal factors by which diabetes increases CV disease have yet to be defined.

T2D accounts for approximately 10% of all vascular deaths in developed countries over the past 10 years [2] and is associated with an increased risk of death from a range of other conditions, including some cancers, infectious diseases, external causes, intentional self-harm, and some degenerative disorders [4]. Data from 97 prospective studies (*n* = 820,900) suggest that, on average, a 50 year old with T2D and no history of vascular disease dies approximately 6 years before their counterpart without T2D [4]. Whereas the risk of death is directly associated with levels of fasting plasma glucose (FPG) in the diabetic range (>5.6 mmol/l; 100 mg/dl), there appears to be only a modest correlation between FPG levels in the nondiabetic range (3.9 to 5.6 mmol/l; 70 to 100 mg/dl) [4]. Similarly, the Emerging Risk Factors Collaboration found that assessment of neither FPG concentration nor impaired fasting glucose status significantly improved vascular disease prediction in people without T2D beyond that obtained from conventional risk factors. Moreover, although both early-onset and late-onset T2D are associated with an increased risk of major CHD events and mortality, only long-duration T2D (>10 years) appears to be a CHD risk equivalent [3]. Together, these studies suggest that the duration of hyperglycaemia might contribute to the association between T2D and vascular risk.

The impact of hyperglycaemia on T2D-related vascular risk was further examined by a meta-analysis of five randomised clinical trials (*n* = 33,040) comparing the effects of intensive versus standard glycaemic control in people with T2D [1]. Compared with standard glycaemic control, intensive control reduced mean glycated haemoglobin A1c (HbA1c) levels by 0.9%, resulting in a 17% reduction in the risk of nonfatal myocardial infarction (odds ratio = 0.83, 95% CI = 0.75 to 0.93) and a 15% reduction in the risk of CHD (odds ratio = 0.85, 95% CI = 0.77 to 0.93) with no significant effect on stroke or all-cause mortality. However, the reduction in events (2.9 per 200 T2D patients treated over 5 years) was far more modest than that achieved either with lipid-lowering therapy (8.2 fewer CV events for each mmol/l reduction in low-density lipoprotein-cholesterol) or with antihypertensive treatment (12.5 fewer events per 4 mmHg reduction in systolic blood pressure) [1]. Similarly, the Diabetes Reduction Assessment with Ramipril and Rosiglitazone Medication (DREAM) study (*n* = 5,269) showed that, although oral hypoglycaemic agents can increase the likelihood of regression to normoglycaemia and can reduce the incidence of T2D in adults with impaired FPG and/or impaired glucose tolerance, they have very little effect on CV event rates [5].

Overall, these results suggest that T2D and dysglycaemia are different entities and that the CV risk associated with dysglycaemia is modest.

## Some statins are associated with an increased risk of new-onset type 2 diabetes

Numerous studies have shown that reducing low-density lipoprotein-cholesterol levels using statins significantly reduces CV risk in people with and without T2D [6-10]. Although statins are generally safe and well tolerated [11], conflicting data exist regarding the effects of some statins on the risk of incident T2D [12-17]. For example, the Justification for the Use of Statins in Primary Prevention: An Intervention Trial Evaluating Rosuvastatin (JUPITER) study (*n* = 17,802) showed a significant 3.0% versus 2.4% increase in incident T2D among healthy adults treated with rosuvastatin 20 mg/day versus placebo for 1.9 years (*P* = 0.01) [12]. This was accompanied by a small but significant increase in HbA1c levels. In contrast, the West of Scotland Coronary Prevention Study (WOSCOPS; *n* = 5,974) showed that, compared with placebo, pravastatin was associated with a 30% reduction (*P* = 0.042) in the hazard of developing T2D after 5 years [13].

**Figure 2:**
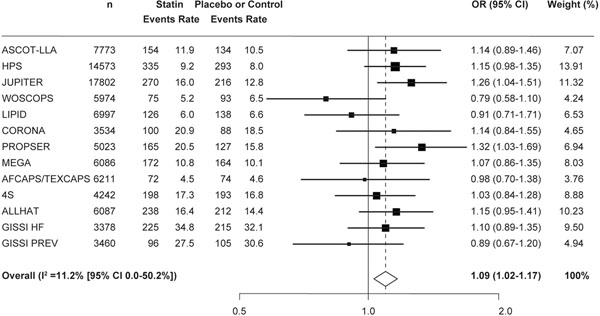
**Statin therapy is associated with an increased risk of type 2 diabetes **[15]**.** Events per 1,000 patient-years; weights are from random-effects analysis. CI, confidence interval; OR, odds ratio.

To investigate this discrepancy, a meta-analysis of 13 statin trials was performed, including 91,140 patients without T2D [15]. In this analysis, statin therapy (atorvastatin 10 mg, pravastatin 40 mg, simvastatin 40 mg or rosuvastatin 20 mg) was associated with a 9% increased risk for T2D over 4 years (odds ratio = 1.09, 95% CI = 1.02 to 1.17), with only a small degree of heterogeneity (*I*^2^ = 11%) between trials (Figure 2). A further meta-analysis showed that the potential diabetogenic effects of statins may be dose related [14]. Compared with moderate therapy, intensive therapy (simvastatin 80 mg or atorvastatin 80 mg) was associated with a higher incidence of T2D (odds ratio = 1.12), with no heterogeneity between trials (*I*^2^ = 0%). Similar results were also obtained from a *post-hoc* analysis of the Stroke Prevention by Aggressive Reduction in Cholesterol Levels (SPARCL) trial (*n* = 3,803) [17]. Incident T2D occurred in 8.71% of patients randomised to atorvastatin 80 mg/ day and in 6.06% of patients receiving placebo (adjusted HR = 1.37, 95% CI = 1.08 to 1.75; *P* = 0.011).

Although these results suggest a correlation between statin therapy and incident T2D, it should be noted that none of the statin trials were designed to look for incident T2D and that the meta-analyses used a range of methods to detect the condition. Further studies are therefore required to fully understand this effect.

## What does this mean for our patients?

The benefits of intensive versus standard statin therapy on CV outcomes in people with and without T2D have been clearly established [6,18-21]. For example, the Treat to New targets (TNT) study (*n* = 10,001) found that the total number of CV events prevented per 5,000 patients treated with atorvastatin 80 mg versus atorvastatin 10 mg for 5 years was 608 (262 prevented for the first event, and 166, 92, 55 and 33 prevented for the second, third, fourth and fifth events) [18]; similar findings were observed in the subgroup of patients with T2D or metabolic syndrome (*n* = 5,854). These results are further corroborated by a meta-analysis carried out by the Cholesterol Treatment Trialist’s Collaboration, in which more versus less intensive statin therapy was associated with a highly significant 15% (95% CI = 11 to 18; *P* <0.0001) further reduction in major vascular events [6]. Importantly, each 1.0 mmol/l decrease in low-density lipoprotein-cholesterol reduced the annual rate of major CV events by 21% in patients both with and without T2D [6,21].

Based on these results [6,21], Sattar and colleagues found that treating 255 patients with standard-dose statin therapy for 4 years would, on average, avoid a composite of nine vascular events whilst leading to one case of statin-related T2D (9:1 benefit vs. risk ratio) [15]. Similarly, Preiss and colleagues found that, compared with standard therapy, the number needed to harm per year for intensive statin therapy was 498 for incident T2D whereas the number needed to treat per year was 155 for CV events [14]. In absolute terms, intensive statin therapy accounted for 3.0 additional cases of T2D and 6.5 fewer first major CV events per 1,000 patient-years.

Together, these studies suggest that the overwhelming benefits for intensive-dose statins in reducing CV events far outweigh the small absolute risk for developing T2D. Authors therefore conclude that statins should still be considered as a first-line therapy for dyslipidaemia in the majority of patients with CV risk.

## Are all statins the same?

Mechanisms explaining the potentially higher incidence of T2D with statin therapy have not yet been identified. Possible explanations include residual confounding factors, such as improved survival with statin treatment and an improved lifestyle after CV events. However, independent predictors for statin-associated T2D appear to include elevated levels of baseline FPG, BMI, blood pressure and fasting triglycerides [17]. Moreover, although some statins have been associated with increased HbA1c levels in patients receiving intensive therapy, other statins have demonstrated neutral or favourable effects on glucose control in patients with and without T2D [16,22-31].

Analysis of data from the PROVE-IT TIMI 22 trial showed that, among the 3,382 patients without preexisting T2D, HbA1c levels increased by 0.12% in patients treated with pravastatin 40 mg and by 0.30% in those receiving atorvastatin 80 mg (*P* <0.0001) [23]. However, these results were obtained from a *post hoc* analysis and must be interpreted with caution. Similarly, a comparison of glycaemic control between T2D patients receiving atorvastatin 10 mg, pravastatin 10 mg or pitavastatin 2 mg/day (*n* = 279) showed that glycaemic parameters (arbitrary blood glucose levels and HbA1c) only increased among atorvastatin-treated patients [24]. Since there was no correlation between changes in HbA1c and changes in low-density lipoprotein-cholesterol, the mechanism of statin-effects on glycaemic control were deemed to be unrelated to lipid profile. Again, these data should be treated with caution due to the retrospective, single-site nature of the study. However, the results are consistent with those from a subanalysis of the CHIBA study in which 45 Japanese patients with T2D and hypercholesterolaemia were randomised to pitavastatin 2 mg or atorvastatin 10 mg for 12 weeks [25]. Whereas atorvastatin was associated with a significant increase in serum glycoalbumin levels (+0.67 ± 1.31% vs. baseline; *P* = 0.026) and a slight increase in HbA1c levels (*P* = 0.098), pitavastatin had no obvious effect.

In contrast to atorvastatin’s negative effects, pitavastatin appears to have a neutral and possibly beneficial effect on glucose regulation. The CHIBA study showed slight increases in FPG, insulin and the homeostasis model assessment ratio with atorvastatin but not pitavastatin [25]. More recently, the CAPITAIN study showed that 6-month treatment with pitavastatin 4 mg did not significantly change the mean FPG, homeostasis model assessment index, insulin levels, insulin/glucose ratios, or HbA1c levels in people with metabolic syndrome [30]. Two further studies showed that 12-week treatment with pitavastatin 4 mg had no effect on FPG levels in patients with primary hyperlipidaemia or mixed dyslipidaemia and ≥2 additional risk factors for CHD [32], and no effect on FPG, fasting plasma insulin, HbA1c, HOMA-IR or QUICKI in patients with primary hyperlipidaemia or mixed dyslipidaemia [33]. Furthermore, a subanalysis of the LIVALO Effectiveness and Safety (LIVES) study showed a significant (*P* <0.001) 0.28% decrease in HbA1c levels among 308 patients with T2D after 104 weeks of pitavastatin treatment [26].

Together these data suggest that, whereas some statins have negative effects on glucose control, pitavastatin has a neutral and possibly beneficial effect. However, much of the evidence derives from relatively small, retrospective and/or single-centre studies and requires confirmation in more robust trials. One such study – the Japan Prevention Trial of Diabetes by Pitavastatin in Patients with Impaired Glucose Tolerance (J-PREDICT) study – is an open-label, randomised controlled, parallel-group comparative study designed to evaluate the cumulative incidence of new-onset T2D in ~1,240 patients with impaired glucose tolerance following 5-year treatment with pitavastatin 1 to 2 mg/day [34]. This study will be completed in 2015.

In conclusion, conflicting data exist regarding the dose-dependent diabetogenic effects of statins. Whereas some statins (for example, atorvastatin, pravastatin, rosuvastatin and simvastatin) appear to have adverse effects on glycaemic control, others (for example, pitavastatin) appear to have a neutral and possibly favourable effect. However, more robust studies are required to confirm these results and to fully understand their clinical implications. In the meantime, the net CV benefit favours the use of statin therapy in patients with dyslipidaemia, irrespective of T2D risk.

## Abbreviations

BMI: body mass index; CHD: coronary heart disease; CI: confidence interval; CV: cardiovascular; FPG: fasting plasma glucose; HbA1c: haemoglobin A1c; HR: hazard ratio; T2D: type 2 diabetes.

## Competing interests

KR received honoraria from Novo Nordisk, Roche, Novartis, Pfizer, Astra Zeneca, Daiichi Sankyo, and Lilly. KR represents the advisory boards of Novo-Nordisk, Roche, Astra Zeneca, Pfizer, Daiichi Sankyo, Lilly, and MERCK.

## Declaration

This article has been published as part of *Cardiovascular Diabetology* Volume 12 Supplement 1, 2013: Statins in cardiometabolic disease: what makes pitavastatin different? The full contents of the supplement are available online at http://www.cardiab.com/supplements/12/S1. Publication of this supplement has been funded by Kowa Pharmaceutical Europe. Pitavastatin is a product marketed by the sponsor of the supplement. Articles are based on the proceedings of the World Congress for the Prevention of Diabetes. Authors received support with the preparation of their articles from GK Pharmacomm, an agency funded by the sponsor. All articles have undergone the journal’s standard peer review process. The Supplement Editors declare that they have no competing interests.
